# Cefiderocol for Treatment of Ventriculitis (4MRGN *A. baumannii*)—Results of Therapeutic Drug Monitoring in Blood and Cerebrospinal Fluid

**DOI:** 10.3390/antibiotics15020139

**Published:** 2026-01-31

**Authors:** Melita Hadzifejzovic, David Guevara Lara, Samir G. Sakka

**Affiliations:** 1 Department of Anesthesia, Emergency and Pain Medicine, Gemeinschaftsklinikum Mittelrhein gGmbH, Ev. Stift St. Martin, Academic Teaching Hospital of the Johannes Gutenberg-University Mainz, D-56068 Koblenz, Germany; melita.hadzifejzovic@gk.de (M.H.); david.guevara@gk.de (D.G.L.); 2 Department of Intensive Care Medicine, Gemeinschaftsklinikum Mittelrhein gGmbH, Ev. Stift St. Martin, Academic Teaching Hospital of the Johannes Gutenberg-University Mainz, D-56068 Koblenz, Germany

**Keywords:** infections, multi-drug resistance, ventriculitis, *Acinetobacter baumannii*, *Klebsiella pneumoniae*, cefiderocol, therapeutic drug monitoring

## Abstract

**Background**: Cefiderocol, a siderophore cephalosporin, is approved for the treatment of infections caused by multi-drug-resistant Gram-negative bacteria (MRGN). At present, few data are available on the pharmacokinetics of this substance in critically ill patients, particularly for the treatment of central nervous system infections. **Patients and Methods**: Here, we reported on a 22-year-old male patient after severe open head trauma. Initial screening revealed colonization with 4MRGN *A. baumannii* (OXA-23) (perianal) and 4MRGN *K. pneumoniae* (KPC) (tracheal). Unfortunately, he developed ventriculitis (4MRGN *A. baumannii*). According to microbiological testing, the patient with normal renal function received 3 × 2 g/d i.v. cefiderocol as a prolonged infusion (3 h) and colistin 3 × 3 Mio. IU/d i.v. for 2 weeks. In addition to serum trough levels, drug monitoring was performed in the cerebrospinal fluid (CSF) via external ventricular drainage (24 h aliquots). **Results**: Serum and CSF specimens analyzed by liquid chromatography–mass spectroscopy (LC-MS) in the presence of severe meningeal inflammation yielded average CSF concentrations of cefiderocol from 5.48 to 8.40 (median 6.98) μg/mL and a concentration ratio C_CSF mean_/C_serum trough_ from 0.38 to 0.76 (median 0.48). The cefiderocol levels in the CSF were sufficient for eradication of *A. baumannii*. A subsequent CSF infection with *K. pneumoniae* (found initially in screening and resistant to cefiderocol) after completed treatment with cefiderocol was successfully treated with gentamicin (intrathecally) and ceftazidime/avibactam (i.v.). However, the patient died due to a *Candida tropicalis* infection detected in the CSF on day 71. **Conclusions**: Our results indicate that standard dosages of cefiderocol are sufficient for treatment of CNS infections in the presence of a severe disruption of the blood–CSF barrier.

## 1. Introduction

Cefiderocol, a siderophore cephalosporin, has been available as a treatment option for patients with multi-resistant Gram-negative bacteria (MRGN), including metallo-β lactamase-expressing strains, since April 2020 (EU) [1]. The active cell entry mechanism via the catechol side chain enhances the stability against β-lactamases. It has been used successfully in complicated urinary tract infections, respiratory tract infections and bloodstream infections [2,3,4].

Infections of the central nervous system pose a particular challenge in intensive care medicine. Ensuring adequate drug levels at the site of infection with the need to pass the blood–brain or blood–cerebrospinal fluid barrier sometimes requires significantly higher doses, as has been shown for meropenem [5,6]. Markers of inflammation can be used clinically as a surrogate for penetration into the cerebrospinal fluid space with several limitations [7]. In view of the complexity and severity of the infection, therapeutic drug monitoring (TDM) is often regarded as necessary in critically ill patients, even though there is no scientific evidence to date that it improves outcomes.

To date, only few data are available on the penetration of cefiderocol into the cerebrospinal fluid (CSF). Colombo et al. [8] reported the successful treatment of a patient with meningitis (*K. pneumoniae* (KP), minimal inhibitory concentration [MIC] < 2 µg/mL) with cefiderocol and fosfomycin. However, antibiotic concentrations in the CSF were not determined. In meningitis caused by *A. baumannii* (carbapenem-resistant), eradication of the pathogen and sufficient concentration in the CSF (CSF trough level 15.9 µg/mL) was demonstrated [9]. These authors also mentioned plasma levels revealing approximate 50% of free substance. In a case series of 17 patients, including 1 case of meningitis caused by multi-resistant *P. aeruginosa* [10], that were treated with a cefiderocol-containing regime, a CSF concentration of 13 µg/mL (plasma 105 µg/mL) was determined. Luque-Paz et al. [11] (in combination with colistin, amikacin) and Stevenson et al. [12] (combined with intrathecal colistin) described successful microbiological eradication (*P. aeruginosa*) for cefiderocol at different doses, with CSF concentrations between 1.22 and 16.80 µg/mL.

In the following, we report on the results of therapeutic drug monitoring (blood and cerebrospinal fluid) of cefiderocol therapy for *A. baumannii* (4MRGN)-associated ventriculitis in routine clinical practice.

## 2. Case Report

During his holidays in Turkey, a 22-year-old man (76 kg, 178 cm) driving a jeep collided with a truck. With a Glasgow Coma Scale (GCS) of 3–4, he was admitted to hospital as an emergency patient, intubated and ventilated. A computed tomography (CT) scan showed severe traumatic brain injury (TBI) with a bilateral subdural hematoma with right frontal and frontobasal contusion hemorrhages. There were grossly dislocated fractures of the left temporal bone with transverse and longitudinal fractures. Furthermore, wedged bony fragments in the longitudinal fracture above the auditory canal involving the upper edge of the cochlea were seen. A transverse and longitudinal fracture of the temporal bone on the right with fracture crossed the carotid canal. Furthermore, there was a transverse fracture through the anterior clivus and oblique fractures through the posterior clivus with radiation into the foramen magnum, as well as a cleft fracture of the occipital bone, which ran out high dorsoparietally, downwards and forwards over the right occipital condyle and the right jugular bulb with radiation into a petrous bone fracture. In addition, there was a small avulsion from the posterior edge of the facies articularis superior of the right atlas. Chest CT revealed lung contusions and right-sided rib fractures. He underwent emergency head surgery, with hematoma relief and craniectomies on both sides. He was transferred to the intensive care unit (ICU), sedated, tracheally intubated and mechanically ventilated. Antibiotic treatment with ceftriaxone i.v. and clarithromycin i.v. initiated. On the following day, the pupils were narrow and isocoric. An awakening trial was unsuccessful. A CT control of the head revealed severe brain damage. While no indication was given in the written report, vancomycine (2 × 1 g) i.v. was added. A transfer close to home was organized. On transfer 1 week after care abroad, the patient was orotracheally intubated, analgo-sedated and mechanically ventilated. The pupils were found to be 2 mm, isocoric and non-responsive to light. The CT scan showed a post-extensive bifrontal craniotomy with partial ligation of the right sphenoidal artery. Clinically, the suspicion of liquorrhea was confirmed by a β-trace protein test. In the initial screening, a perianal swab was positive for a 4MRGN *A. baumannii* with detected expression of the class-D carbapenemase OXA-23. In parallel, a 4MRGN *K. pneumoniae* (KPC) was isolated from the tracheal canula and confirmed using a tracheal secretion a few days later (Table 1). The patient did not receive antibiotics at this time point. As indicated by laboratory chemistry (i.e., IL-6), a CSF infection was suspected.

**Figure 1:**
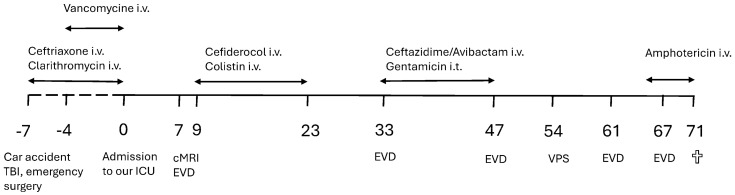
Timeline (days) with relevant time points during the clinical course. ICU: intensive care unit, cMRI: cerebral magnetic resonance tomography imaging, EVD: external ventricular drainage, TBI: traumatic brain injury, VPS: ventriculo-peritoneal shunt, i.t.: intrathecally, white cross: Exitus letalis.

While having been weaned from the respirator, the patient developed a fever up to 39.2 °C on day 5. Cerebral magnetic resonance imaging on day 7 revealed an intraventricular empyema based on left-sided mastoiditis with an empyema/abscess and leptomeningitis with questionable adventitis of the basilar artery. The patient immediately underwent surgical revision and placement of an external ventricular drainage (EVD). Based on microbiological testing of the CSF taken intra-operatively (exclusively 4MRGN *A. baumannii*) and reported two days later, treatment with cefiderocol (3 × 2 g/d i.v. over 3 h) and colistin 3 × 3 Mio. IU/d i.v. (normal renal function) was initiated according to the antibiogram (Table 2). Immediately prior to the start of treatment, a further specimen confirmed *A. baumannii* in the CSF by culture (time to positivity 1.39 h) with an identical resistance profile. After 2 weeks of antibiotic therapy, the first control tests for *A. baumannii* were negative. Ten days after the end of this therapy, 4MRGN *K. pneumoniae* (initially found during screening and resistant to cefiderocol) was detected in the CSF; therefore, ceftazidime/avibactam (i.v.) and gentamicin (intrathecally) were administered. After 2 weeks of therapy, no pathogen was detected in three consecutive CSF samples on days 50–52 after admission. On day 54 after admission to our ICU, a ventriculo-peritoneal (VP) shunt was implanted. On days 64 and 66, *Candida tropicalis* was detected in the CSF; therefore, intravenous treatment with liposomal amphotericin B was initiated. The VP shunt was removed and another EVD was inserted. Unfortunately, in addition to the CNS infection (Table 3), a hemorrhage subsequently developed in the vicinity of the EVD. After detailed discussions with the family, the EVD was removed in view of the severity of the disease, and a palliative treatment concept was defined. Finally, the patient died on day 71 after admission to our ICU. A timeline of the clinical course is provided in Figure 1.

## 3. Materials and Methods

Drug administration: The patient received cefiderocol (Shionogi GmbH, Berlin, Germany) 3 × 2 g per day for 14 days as a prolonged infusion (3 h) via a central venous catheter.

Sample collection and analysis: The serum samples (trough levels) were taken before the 8:00 a.m. administration of cefiderocol. The CSF samples were taken at the same time as a 5 mL aliquot from the CSF collected over 24 h after gentle shaking of the reservoir bag. Whole-blood samples were collected via a catheter placed at a safe distance from the cefiderocol administration site and filled into appropriate tubes (serum monovette, Sarstedt, Nuembrecht, Germany). The samples were transported to the laboratory (MVZ Labor Koblenz, Koblenz, Germany) immediately after collection. After centrifugation (4000 rpm, 10 min at 20 °C), the serum was frozen at −80 °C in the MVZ Labor Koblenz. The samples were collected and sent frozen on dry ice by courier to the Institute for Biomedical and Pharmaceutical Research (IBMP), Nuremberg-Heroldsberg, Germany. The LC-MS analytical method was performed as previously published [12]. The cefiderocol concentration was always measured as the total concentration.

Microbiological testing: Microbiological diagnostics and testing were carried out at the MVZ laboratory in Koblenz. As the MIC was not obtained, the susceptibility for cefiderocol was tested using a disk diffusion test. According to EUCAST (The European Committee on Antimicrobial Susceptibility Testing) in 2024, zone diameters of ≥17 mm for a cefiderocol 30 µg disk correspond to MIC values below the PK-PD breakpoint of S ≤ 2 µg/mL. Results of the microbiological testing are listed in Table 1.

## 4. Results

The laboratory data from the clinical routine (MVZ Labor Koblenz) regarding blood and cerebrospinal fluid diagnostics and the therapeutic drug monitoring of cefiderocol (IBMP Nuremberg-Heroldsberg) are summarized in Table 2. The CSF parameters protein, lactate, leukocytes and the cytokine IL-6 suggest severe meningeal inflammation accompanied by a strong disruption of the blood–CSF barrier.

The average CSF concentrations (C_CSF mean_) ranged from 5.48 to 8.40 (median 6.98) μg/mL, the trough serum concentrations (C_serum trough_) ranged from 7.45 to 20.2 (median 16.2) μg/mL, and the concentration ratio C_CSF mean_/C_serum trough_ ranged from 0.38 to 0.76 (median 0.48). The CSF/blood concentration ratio, as calculated, may be regarded and is used as an indicator for the penetration rate.

## 5. Discussion

The treatment of multi-resistant pathogens, e.g., MDR *A. baumannii,* in critically ill patients poses a particular challenge. Infections of the central nervous system, e.g., ventriculitis, are particularly difficult to treat, as sufficient penetration into the cerebrospinal fluid is required. In the context of an inflammatory meningeal reaction, the penetration rate (CSF/blood), which is physiologically <10% for cephalosporins and <20% for carbapenems [13], may increase. In animal models, it was shown that the penetration rates for cefiderocol are comparable with those of other antibiotics such as piperacillin, cefepime and meropenem. In severely inflamed meninges, a 3-fold increase in the cerebrospinal fluid concentration was demonstrated for cefiderocol in animal experiments [14]. Clinical data for the CSF penetration of cefiderocol is limited. Colombo et al. [8] published the successful treatment of ventriculitis caused by KPC-forming *K. pneumoniae* with 4 × 2 g/d cefiderocol and fosfomycin; unfortunately, no cefiderocol levels in the CSF were determined in this paper. Recent case reports, albeit without CSF level determinations, also confirm successful treatment with cefiderocol in ventriculitis induced by multi-resistant *A. baumannii* [15,16]. Luque-Paz et al. [11] described a case in which a mean serum concentration of 39.0 ± 4.9 µg/mL and a CSF concentration of 16.8 ± 3.1 µg/mL were achieved after a daily dose of 8 g of cefiderocol for several days, followed by a reduction to 6 g/d. This patient with *P. aeruginosa*-associated ventriculitis had also received 10 mg colistin and 30 mg amikacin intrathecally once daily. It should be mentioned that this patient also developed fungal meningitis (*Candida tropicalis*, antifungal treatment not mentioned) and ultimately died. The authors noted that sufficient cefiderocol levels were built up in the CSF (>4 times the MIC). Stevenson et al. [12] described a patient with nosocomial ventriculitis caused by NDM-1-producing *P. aeruginosa*. The pathogen was successfully eliminated from the CSF (in combination with intrathecal and intravenous administration of colistin). The TDM yielded CSF concentrations for cefiderocol between 1.22 and 3.90 µg/mL. The patient described was initially treated with 3 × 1.5 g/d because of renal failure and then 3 × 2 g cefiderocol, as their renal function improved. Marcelo et al. [17] described the use of cefiderocol (4 × 2 g/d) for the treatment of *P. aeruginosa*-related ventriculitis and found concentrations between 1.6 and 3.6 μg/mL in the cerebrospinal fluid. In combination with colistin, which was initially administered intrathecally and later i.v., the pathogen could be eradicated. In their case series, Meschiari et al. [10] reported the use of cefiderocol in a patient with nosocomial ventriculitis drainage meningitis (pathogen: *P. aeruginosa*). Cefiderocol serum levels were measured immediately before administration (−0.25 h) and at the end of the three-hour infusion, resulting in concentrations of 105 µg/mL (C_min_) and 170 µg/mL (C_max_). CSF levels (13 mg/L) were measured 25 min prior to the cefiderocol administration, concurrent with serum trough levels, resulting in a C_min CSF_/C_serum_ ratio of 12.4%. It is important to note that the patient had moderate renal dysfunction (creatinine clearance 44.8 mL/min) and was treated with a high dose of cefiderocol (2 g every 6 h over a 3 h infusion) to optimize drug penetration into the CSF, with no side effects despite the high dosage. An *A. baumannii*-associated ventriculitis treated with 4 × 2 g/d cefiderocol was published by Finch et al. [18]. The authors found serum concentrations between 24.6 and 76.7 μg/mL, while the CSF concentrations were approximately 10.0 μg/mL [18]. This group observed minimal variations in the CSF cefiderocol concentrations, suggesting slow elimination from the CSF compartment. Their experience suggests that a single CSF concentration (random or trough) could be directly compared with the minimum inhibitory concentration of the causative bacterium.

Our own results, obtained in a patient without renal dysfunction, cannot be fully compared with the data available in the literature. Our data does not allow for determination of the area under the curve (AUC) of the serum concentration–time curve. The level of cefiderocol concentration in the cerebrospinal fluid is within the range of previous studies. Based on the CSF concentration averaged over 24 h and the serum trough level, a median C_CSF mean_/C_serum trough_ ratio of almost 50% was attained. This value overestimates the actual penetration rate, as the average CSF concentration over 24 h was divided by the trough serum concentration, i.e., the AUC in serum is unknown, and the true denominator is larger than C_serum trough_ × 24 h. Kufel et al. [9] described a rate of 42% [9]. This working group showed an AUC for the CSF of 146.49 (dose 4 × 2 g/d) or 118.28 µg × h/mL (dose 3 × 2 g/d). Our data for 3 × 2 g/d cefiderocol suggests AUC_CSF_ values between 201.60 µg × h/mL (24 h × 8.40 µg/mL) and 131.52 (24 h × 5.48 µg/mL) µg × h/mL. The probable reason for the excellent CSF penetration in our case is the strong disturbance of the blood–CSF barrier, as indicated by the high CSF protein content (normal: ≤0.45 g/L) and the elevated CSF inflammatory parameters (see Table 2). Although complete concentration–time curves of cefiderocol in CSF have not been published, physicochemical properties suggest that cefiderocol (molecular mass 752.2 g/mol, log partition coefficient −2.265; https://pubchem.ncbi.nlm.nih.gov/compound/Cefiderocol#section=LogP&fullscreen=true (accessed on 27 January 2026)) penetrates the blood–CSF and blood–brain barriers slightly less readily than other β-lactam antibiotics with a smaller size (usually <600 g/mol) [19]. Our data unfortunately do not permit the estimation of the elimination half-life in CSF. Our data also do not permit calculating the ratio C_CSF_/MIC since the exact MIC of the *A. baumannii* strain was not determined in the microbiological routine laboratory. CSF concentrations ≥ 10× the MIC (even better: the minimum bactericidal concentration, MBC) are required for rapid bacterial killing in CNS infections [20]. However, as several antibiotics administered before having limited or no penetration into the CSF, we would like to emphasize the effects of cefiderocol in the CSF. It should be noted that the determination of cefiderocol concentrations began on the sixth day of treatment, when, due to the decrease in inflammation in the CSF, the concentration/level of substance in the CSF may be lower.

Recently, the first data from pediatrics have been published. In a 1-month-old infant, a penetration rate into the cerebrospinal fluid of 69.5% was described [21]. These authors report an AUC in serum (8 h period) of 326.62 µg × h/mL. Unfortunately, a direct comparison of these results with our data is not possible. As we can only provide serum trough concentrations, no reliable statements on the AUC_serum_ can be made. A detailed analysis would have required a much more complex clinical management. Special procedures, such as a simulation of the concentration–time curves, represent possibilities to obtain this data approximately. A retrospective study in 31 critically ill patients using TDM results obtained from a real-world setting revealed that recommendations on dosing of cefiderocol ensured high rates of target attainment, even in patients with renal replacement therapy and a high body mass index [22].

Finally, several potential limitations of our report need to be addressed. As the patient’s muscle weight might have decreased during the ICU treatment, assessment of the renal function, as estimated from serum creatinine or its clearance, may be inaccurate. Furthermore, as we took CSF aliquots from liquor sampled over a 24 h period for lab analysis, and given the stability of cefiderocol over such time intervals is limited [23], our results may have been influenced by this factor.

In general, it is worth mentioning that screening on admission found 4MRGN *K. pneumoniae*, which, though initially not present in the CSF, later caused a CNS infection, emphasizing the value of screening in patients with risk factors.

## 6. Conclusions

Our data suggests that the dosage of cefiderocol in the treatment of ventriculitis (4MRGN *A. baumannii*) in combination with the systemic administration of colistin was sufficient. Unfortunately, we did not obtain colistin concentrations, neither in blood nor in the CSF, and thus, we cannot rule out that colistin was responsible for the eradication. The clinical importance of therapeutic drug monitoring should be emphasized, especially in patients with infections caused by pathogens with multi-drug resistance, to increase the probability of successful treatment when the number of treatment options is low.

## Figures and Tables

**Table 1:** Antibiogram. Detection of *K. pneumoniae* at the tracheal cannula. MIC: minimal inhibitory concentration.

Antibiotic	MIC [mg/L]	Interpretation
Ampicillin	>16	R
Piperacillin	>64	R
Ampicillin/Sulbactam	>16	R
Imipenem	>8	R
Meropenem	>8	R
Ertapenem	2	R
Cefuroxime i.v.	>32	R
Cefotaxime	>32	R
Ceftazidime	>32	R
Cefiderocol		R
Gentamicin	≤1	S
Ciprofloxacin	>2	R
Cotrimoxazole	>160	R

Values for concentrations (numbers) are given in mg/L. MIC: minimal inhibitory concentration. R: resistant, S: susceptible. Cefiderocol susceptibility was assessed using the disk diffusion method.

**Table 2:** Antibiogram. Detection of *A. baumannii* in the cerebrospinal fluid. MIC: minimal inhibitory concentration.

Antibiotic	MIC [mg/L]	Interpretation
Ampicillin		R
Piperacillin		R
Ampicillin/Sulbactam		R
Imipenem	>8	R
Meropenem	>8	R
Cefuroxime i.v.		R
Cefotaxime		R
Ceftazidime		R
Cefiderocol		S
Gentamicin	>8	R
Ciprofloxacin	>2	R
Tigecycline	1	R
Cotrimoxazole	>160	R
Colistin	2	S *

No detection of obligatory aerobic pathogens. Values for concentrations (numbers) are given in mg/L. MIC: minimal inhibitory concentration. R: resistant, S: susceptible. Cefiderocol susceptibility was assessed using the disk diffusion method. * EUCAST recommendation: When used for treatment, then only in combination with another antibiotic substance.

**Table 3:** Laboratory data from clinical routine regarding blood and CSF diagnostics and therapeutic drug monitoring for cefiderocol. Abbreviations: CSF: cerebrospinal fluid, CRP: C-reactive protein, eGFR: estimated (calculated) glomerular filtration rate according to Cockroft–Gault, C_cefiderocol_: serum cefiderocol trough level.

Day	CSF						Serum/Blood					
	Protein [g/L]	Glucose [mg/dL]	Lactate [mmol/L]	IL-6 [pg/mL]	Cell Count [/µL]	C_cefiderocol_ [µg/mL]	Leukocyte Count [/nL]	CRP [mg/dL]	Creatinine [mg/dL]	eGFR [mL/min]	C_cefiderocol_ [µg/mL]	C_CSFmean/Cserum trough_
0	2.59	52	10.88	49,985.0	1702		12.4	18.1	0.70	133.02		
1							14.0	16.2	0.70	133.02		
2							13.5	9.5	0.60	141.72		
3							15.9	7.4	0.70	133.02		
4	5.77	50	7.73	24,641.5	253		12.1	5.8	0.60	141.72		
5							11.3	3.8	0.60	141.72		
6						6.24	9.8	3.4	0.60	152.75	16.30	0.38
7	1.91	63	5.53	4678.0	180	5.63	9.6	2.3	0.50	152.75	7.45	0.76
8						6.98	10.8	1.8	-	-	12.10	0.58
9						7.26	10.5	-	-	-	-	-
10	2.71	56	5.23	3338.0	429	8.40	7.0	2.0	0.50	152.75	17.20	0.49
11						5.48	7.7	1.8	0.50	152.75	20.20	0.27
12						6.84	4.5	1.8	0.50	152.75	16.00	0.43
13						7.67	7.1	1.6	0.50	152.75	16.60	0.46
14						7.71	4.1	2.3	0.50	152.75	14.60	0.53
16	0.61	63	3.44	398.6	37	-	3.4	1.7	0.50	152.75	-	-
17	0.39	59	3.81	231.5	20	-	-	-	-	-	-	-
18	0.37	65	3.38	358.5	5	-	4.8	2.5			-	-

Reference ranges (CSF): Protein 0.15–0.45 g/L, glucose 40–70 mg/dL, lactate 1.10–2.42 mmol/L, leukocytes 0–4/μL. CRP: <0.5 mg/dL, leukocyte count: 3.7–9.2/nL. -: no data available.

## Data Availability

The data are stored digitally locally and are available on request.
